# Role and mechanism of the action of angiopoietin-like protein ANGPTL4 in plasma lipid metabolism

**DOI:** 10.1016/j.jlr.2021.100150

**Published:** 2021-11-18

**Authors:** Sander Kersten

**Affiliations:** Nutrition, Metabolism and Genomics Group, Division of Human Nutrition and Health, Wageningen University, Wageningen, The Netherlands

**Keywords:** triglycerides, lipoprotein lipase, ANGPTL4, ANGPTL8, ANGPTL3, liver-specific inactivation, HDL-C levels, hepatocytes, loss-of-function mutants, pharmacological target, acLDL, acetylated LDL, ANGPTL, angiopoietin-like protein, BAT, brown adipose tissue, LOF, loss of function, oxLDL, oxidized LDL, PCSK3, proprotein convertase subtilisin kexin-3, WAT, white adipose tissue

## Abstract

Triglycerides are carried in the bloodstream as the components of very low-density lipoproteins and chylomicrons. These circulating triglycerides are primarily hydrolyzed in muscle and adipose tissue by the enzyme lipoprotein lipase (LPL). The activity of LPL is regulated by numerous mechanisms, including by three members of the angiopoietin-like protein family: ANGPTL3, ANGPTL4, and ANGPTL8. In this review, we discuss the recent literature concerning the role and mechanism of action of ANGPTL4 in lipid metabolism. ANGPTL4 is a fasting- and lipid-induced factor secreted by numerous cells, including adipocytes, hepatocytes, (cardio)myocytes, and macrophages. In adipocytes, ANGPTL4 mediates the fasting-induced repression of LPL activity by promoting the unfolding of LPL, leading to the cleavage and subsequent degradation of LPL. The inhibition of LPL by ANGPTL4 is opposed by ANGPTL8, which keeps the LPL active after feeding. In macrophages and (cardio)myocytes, ANGPTL4 functions as a lipid-inducible feedback regulator of LPL-mediated lipid uptake. In comparison, in hepatocytes, ANGPTL4 functions as a local inhibitor of hepatic lipase and possibly as an endocrine inhibitor of LPL in extra-hepatic tissues. At the genetic level, loss-of-function mutations in ANGPTL4 are associated with lower plasma triglycerides and higher plasma HDL-C levels, and a reduced risk of coronary artery disease, suggesting that ANGPTL4 is a viable pharmacological target for reducing cardiovascular risk. Whole-body targeting of ANGPTL4 is contraindicated because of severe pathological complications, whereas liver-specific inactivation of ANGPTL4, either as monotherapy or coupled to anti-ANGPTL3 therapies might be a suitable strategy for lowering plasma triglycerides in selected patient groups. In conclusion, the tissue-specific targeting of ANGPTL4 appears to be a viable pharmacological approach to reduce circulating triglycerides.

Lipoproteins are the main carriers of lipids in the bloodstream. Cholesterol is mainly transported in low-density lipoproteins (LDL) and high-density lipoproteins (HDL), whereas triglycerides (TG) are carried through the bloodstream in the triglyceride-rich lipoproteins chylomicrons and very low-density lipoproteins. The hydrolysis of circulating TG in adipose tissue, heart, and skeletal muscle is catalyzed by the enzyme lipoprotein lipase (LPL) (1). LPL is produced in adipocytes and (cardio)myocytes and is transported and attached to the capillary surface by the endothelium-derived protein GPIHBP1 (2). Consequently, mutations in LPL and GPIHBP1 are associated with hypertriglyceridemia as a result of impaired LPL-mediated TG hydrolysis (3, 4).

Because LPL is rate-limiting for TG uptake into tissues, the activity of LPL in the different tissues is carefully regulated to match the release and uptake of TG-derived fatty acids to the local tissue demand (5, 6). In the last 2 decades, our understanding of how the activity of LPL is regulated has improved dramatically. Two groups of circulating proteins are mainly involved in regulating LPL. The first group is composed of the apolipoproteins C1, C2, C3, E, and A5, whereas the second group consists of three members of the angiopoietin-like protein family (ANGPTL): ANGPTL3, ANGPTL4, and ANGPTL8 (5, 6).

ANGPTL4 was discovered independently by three groups in 2000 (7, 8, 9). These initial papers suggested a link between ANGPTL4 and lipid metabolism and found that ANGPTL4 is transcriptionally controlled by PPARs. However, no distinct functional role was assigned to ANGPTL4 at that time. Shortly thereafter, Yoshida *et al.* demonstrated that ANGPTL4 and the highly related protein ANGPTL3 can inhibit LPL and raise plasma TG levels (10, 11). *ANGPTL3* expression is restricted to the liver, whereas *ANGPTL4* is expressed in numerous tissues and cells, including liver, adipose tissue, kidney, intestine, heart, skeletal muscle, macrophages, and cancer cells (12). Both proteins are composed of an N-terminal signal peptide, an N-terminal coiled-coil domain that contains the LPL-binding region, a linker region, and a C-terminal fibrinogen-like domain. ANGPTL8 was discovered in 2012 as a truncated angiopoietin-like protein that lacks the C-terminal fibrinogen-like domain (13, 14, 15). Its main function seems to be the enhancement or suppression of LPL inhibition by ANGPTL3 and ANGPTL4, respectively, through the formation of a complex (16, 17, 18, 19).

The impact of ANGPTL4 on plasma lipid levels in humans is very well supported by genetic data, showing that the carriers of loss-of-function (LOF) variants in ANGPTL4 have lower plasma TG and higher plasma HDL-C levels than noncarriers (20, 21, 22, 23). The most common genetic variant is E40K, which gives rise to the production of an unstable ANGPTL4 protein (24). The frequency of heterozygous individuals varies from 0.1% in African Americans to a reported 18% in a Tunisian population (25, 26). E40K carrier status was found to be associated with a significantly reduced risk of coronary artery disease (20, 21, 27, 28), suggesting that the inactivation of ANGPTL4 may be a viable pharmacological strategy to improve plasma lipid levels and reduce the risk of coronary artery disease.

In this review, I will discuss the most recent experimental and mechanistic studies on the functional role of ANGPTL4 in lipid metabolism in various tissues. The studies that have tied ANGPTL4 to other (nonmetabolic) biological processes will not be reviewed here.

## Physiological role of ANGPTL4 in WAT

### Role of ANGPTL4 in LPL regulation during feeding and fasting

LPL is rate-limiting for the postprandial storage of circulating TG and functions as a critical mediator in the stimulation of TG storage by insulin (1). Consistent with this role, the activity of LPL in white adipose tissue is high in the fed state and low after prolonged fasting (29). Most of the regulation of LPL activity is thought to occur at the posttranslational level. Indeed, the mRNA levels of *LPL* in adipose tissue differ little between the fed and fasted state (30). Instead, fasting is associated with an increased rate of degradation of LPL within the Golgi/postGolgi secretory compartment and the conversion of active LPL into inactive LPL (31, 32). This shift to inactive LPL is dependent on switching on a gene other than *LPL* (33). It is now evident that the identity of this fasting-induced gene is *ANGPTL4*, originally referred to as the fasting-induced adipose factor FIAF (7). ANGPTL4 is expressed at high levels in mouse and human adipose tissue, both at the mRNA and protein level (12, 30, 34, 35, 36).

**Fig. 1:**
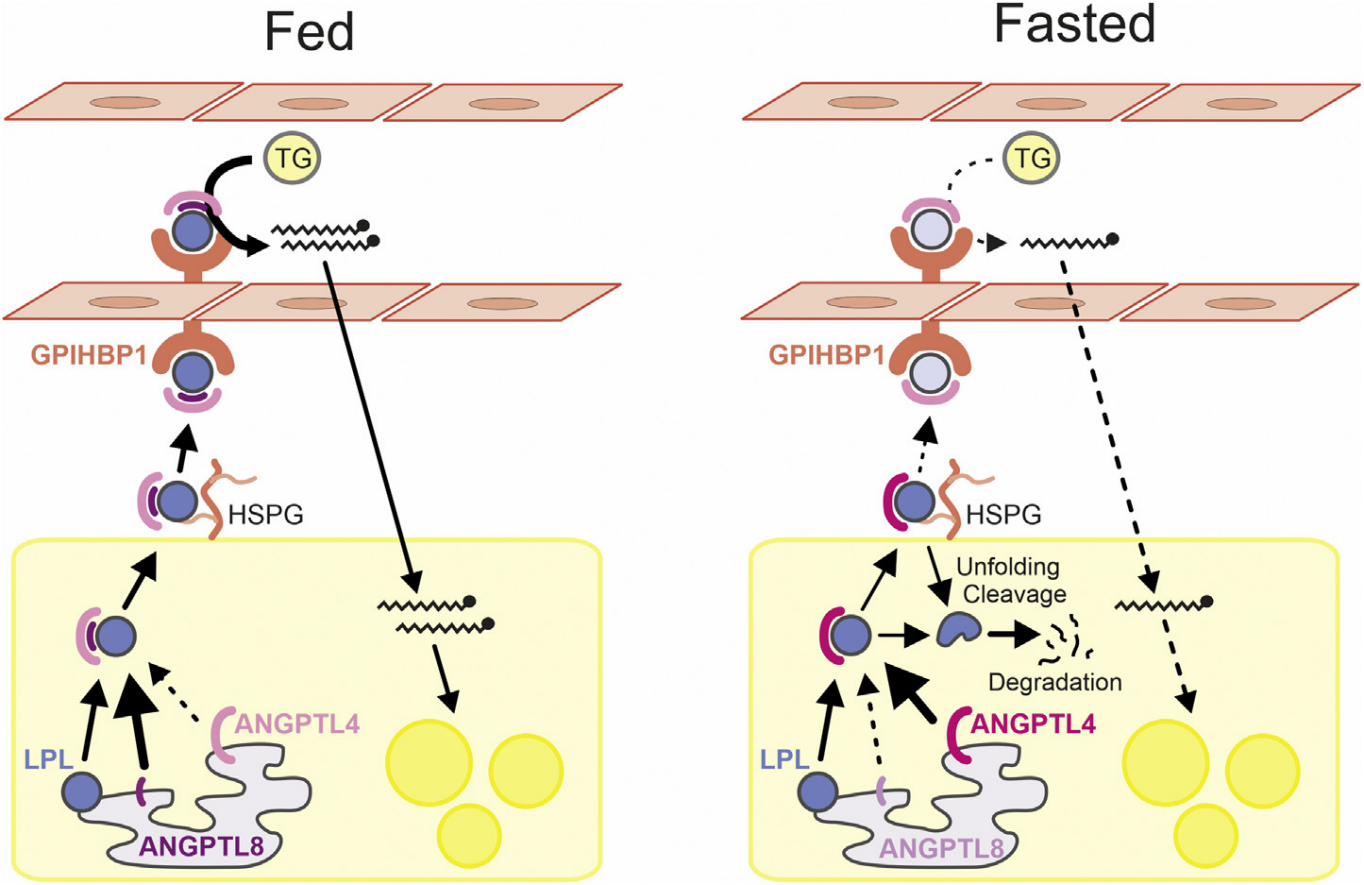
Cartoon depicting the role of ANGPTL4 in LPL regulation in adipose tissue. The relative color intensities of the symbols for LPL, ANGPTL4, and ANGPTL8 reflect their relative protein abundance. In the fed state, ANGPTL8 levels in adipocytes are high, and ANGPTL4 levels are low. ANGPTL8 binds to ANGPTL4, impairing the ability of ANGPTL4 to inhibit LPL and/or promoting ANGPTL4 degradation. As a consequence, the LPL activity and uptake of plasma TG-derived fatty acids in adipose tissue are high. In the fasted state, ANGPTL4 levels in adipocytes are high, and ANGPTL8 levels are low. ANGPTL4 interacts with LPL in adipocytes and/or on the cell surface, triggering the unfolding of LPL, which in turn leads to PCSK3-mediated cleavage and further degradation of LPL. As a consequence, very little LPL and ANGPTL4 might make it to the luminal surface of adipose capillaries in the fasting state, which is reflected by lighter color intensities of the LPL and ANGPTL4 symbols. Concomitant with reduced delivery of LPL to the capillary surface, the LPL activity and uptake of TG-derived fatty acids in adipose tissue are low, thereby directing circulating TG to other tissues. ANGPTL, angiopoietin-like protein; PCSK3, proprotein convertase subtilisin kexin-3.

Overexpression and inactivation studies in genetically modified mice have demonstrated a crucial role of ANGPTL4 in LPL regulation. In the fasted but not the fed state, whole-body overexpression of ANGPTL4 raises plasma TG levels, reduces clearance of plasma TG, and reduces the uptake of plasma TG-derived fatty acids into adipose tissue (37, 38). Conversely, whole-body inactivation or partial inactivation of ANGPTL4 decreases plasma TG levels, increases adipose tissue LPL activity, enhances the clearance of plasma TG, and enhances uptake of plasma TG-derived fatty acids into adipose tissue, which again is especially observed in the fasted but not the fed state (39, 40, 41, 42, 43). Studies in adipocyte-specific ANGPTL4-deficient mice underscore the suppressive effect of adipocyte-derived ANGPTL4 on adipose LPL activity and plasma TG clearance (44). The increase in adipose LPL activity in ANGPTL4-deficient mice is accompanied by an increase in adipose LPL mass, representing the EndonucleaseH-resistant LPL within the Golgi/postGolgi, and a decrease in the N-terminal LPL fragment (43, 45, 46). The latter is a product of LPL cleavage catalyzed by the enzyme proprotein convertase subtilisin kexin-3 (PCSK3) (46). ANGPTL4 reduces LPL mass within the Golgi/postGolgi compartment of adipocytes by promoting PCSK3-mediated LPL cleavage and subsequent LPL degradation (46). The cleavage of LPL by PCSK3 is triggered by the ANGPTL4-induced unfolding of LPL (46, 47). Taken together, the collective data indicate that in white adipose tissue ANGPTL4 primarily functions as an autocrine regulator of LPL during fasting by promoting LPL degradation, thereby reducing the amount of active endothelial-bound LPL participating in TG hydrolysis (Fig. 1).

Studies in human volunteers support this model. Prolonged fasting leads to a decrease in LPL activity and LPL mass in human adipose tissue, concomitant with an increase in ANGPTL4 mRNA and protein (30, 48). Furthermore, in a cross-sectional analysis of human subcutaneous adipose tissue biopsies, ANGPTL4 and LPL protein levels were negatively correlated (35).

Consistent with the important role of ANGPTL4 in adipose lipid metabolism during fasting, mRNA and protein levels of ANGPTL4 are increased in the fasted state in mouse and human adipose tissue (7, 30, 46). Upregulation of ANGPTL4 mRNA and protein by fasting is likely mediated by a combination of reduced insulin signaling, increased plasma glucocorticoids, and elevated free fatty acids (30, 49, 50).

Genetic support for the notion that ANGPTL4 specifically regulates plasma TG in the fasted state is provided by data showing that carrier status of a truncation variant of ANGPTL4 was only associated with lower plasma TG when individuals were fasted for at least 4 h (23). Collectively, it is evident that induction of ANGPTL4 mediates the decrease in LPL content and activity in adipose tissue during fasting, thereby partitioning circulating TG to other tissues (44).

### ANGPTL4 function is antagonized by ANGPTL8

Recent studies indicate that the inhibitory action of ANGPTL4 toward LPL is opposed by locally produced ANGPTL8 through the formation of a protein complex (16, 19). ANGPTL4 alone potently inhibits LPL, whereas the complex of ANGPTL4 and ANGPTL8 is a much weaker inhibitor of LPL (16, 19). It has been suggested that the ANGPTL4/8 complex may shield LPL from inhibition by the circulating complex of ANGPTL3 and ANGPTL8 (16). Other data suggest that via intracellular binding of ANGPTL8 to ANGPTL4, ANGPTL8 may promote the degradation of ANGPTL4 in adipocytes (51). Regardless of the specific mechanism, the inhibitory action of ANGPTL8 toward ANGPTL4 contributes to the elevated adipose LPL activity in the fed state, thereby facilitating the uptake of TG into adipose tissue rather than muscle (16, 51).

### ANGPTL4 stimulates lipolysis in adipocytes

Besides elevating plasma TG levels, recombinant ANGPTL4 injection and transgenic ANGPTL4 overexpression raise plasma nonesterified fatty acid levels (11, 38). Conversely, ANGPTL4 deficiency abolishes the fasting-induced increase in plasma nonesterified fatty acids (43, 52, 53). In cultured adipocytes, ANGPTL4 stimulates fatty acid release concomitant with an increase in cAMP levels (52, 53). ANGPTL4 modulation of cAMP-dependent signaling occurs upstream of adenylate cyclase and downstream of receptor activation and is reportedly mediated by the C-terminal fibrinogen-like domain of ANGPTL4 (52, 54). In adipose tissue, the combined action of the N-terminal and C-terminal domain of ANGPTL4 importantly contributes to the fasting-induced shift from TG storage to TG mobilization.

## Physiological role of ANGPTL4 in brown adipose tissue

LPL is expressed at high levels in brown adipose tissue (BAT), where it accommodates the high requirement of BAT for lipid fuels during cold (55). In agreement with this notion, the activity of LPL in BAT is markedly increased by cold exposure (34). This effect is at least partly mediated by the downregulation of ANGPTL4. Indeed, overexpression of ANGPTL4 leads to decreased LPL activity and uptake of plasma TG-derived fatty acids into BAT, whereas ANGPTL4 deficiency has the exact opposite effect. The downregulation of ANGPTL4 in BAT by cold was found to be mediated by the activation of 5' adenosine monophosphate-activated protein kinase (34).

In contrast to BAT, ANGPTL4 is upregulated by cold in white adipose tissue (WAT), which is likely mediated by β-adrenergic activation. The opposite regulation of ANGPTL4 in BAT and WAT contributes to the repartitioning of lipid fuels toward BAT and away from WAT during prolonged cold exposure (34). Similar to the situation in WAT in the fed state, the upregulation of ANGPTL8 expression in BAT by cold may interfere with ANGPTL4 function, thereby stimulating LPL activity (19, 56).

## Physiological role of ANGPTL4 in the liver

*ANGPTL4* mRNA is expressed at high levels in the human liver (12, 36, 57). Furthermore, it was found that the levels of ANGPTL4 protein are similar in the human liver and human adipose tissue (58). Expression of *ANGPTL4* in normal and hepatocyte-humanized mouse liver is upregulated by fasting, suggesting that the role of hepatic ANGPTL4 in lipid metabolism may become more prominent during fasting (7, 59). Earlier studies indicated that the liver-specific overexpression of ANGPTL4 raises plasma TG levels and decreases postheparin plasma LPL activity but does not influence hepatic VLDL secretion (24, 41, 60). More recent studies confirm the stimulatory effect of hepatocyte-derived ANGPTL4 on plasma TG by showing that the hepatocyte-specific deficiency of ANGPTL4 lowers plasma TG levels in the prolonged fasted state, but not after a 6 h fast (57, 61). Interestingly, Spitler *et al.* (61) did not find any significant effect of hepatocyte-specific ANGPTL4 deficiency on uptake of plasma TG-derived fatty acids by the liver and other tissues, as well as no effect on hepatic-lipase activity in the liver and LPL activity in muscle and adipose tissue. By contrast, Singh *et al.* (57) observed that hepatocyte-specific ANGPTL4-deficient mice exhibited higher uptake of plasma TG-derived fatty acids by the liver and higher post-heparin plasma hepatic lipase and LPL activity. Besides by differences in the types of measurements performed, these seemingly discrepant results might be partly reconciled by differences in the duration of fasting, suggesting that hepatic ANGPTL4 is primarily or exclusively involved in the regulation of plasma lipids during (prolonged) fasting. Consistent with this notion, the silencing of *ANGPTL4* mRNA using antisense oligonucleotides significantly reduced plasma TG levels in overnight fasted mice (57). Collectively, these data show that hepatic ANGPTL4 raises plasma TG in the (prolonged) fasted state. Currently, it is not fully clear whether these effects are mediated by the inhibition of LPL in extra-hepatic tissues via an endocrine role of hepatocyte-derived ANGPTL4 or by inhibition of hepatic lipase via a local role of ANGPTL4 in the liver. In favor of the latter mechanism, hepatocyte-specific deficiency of ANGPTL4 markedly increased postheparin plasma hepatic lipase activity, whereas the overexpression of ANGPTL4 decreased postheparin hepatic lipase activity (37, 57).

The expression of *LPL* in human liver is very low, whereas evidence has been presented that *LPL* mRNA expression is not insignificant in the mouse liver (62). Interestingly, hepatic LPL was shown to significantly impact plasma TG levels and postheparin plasma LPL activity using two different gene targeting approaches (62). Accordingly, it cannot be fully excluded that the plasma TG-raising effect of liver-derived ANGPTL4 in mice is mediated by the inhibition of hepatic LPL. Because of the minimal expression of LPL in the human liver, this mechanism is probably irrelevant in humans.

## Physiological role of ANGPTL4 in other tissues

### Role of ANGPTL4 in the heart and skeletal muscles

ANGPTL4 expression in the heart and skeletal muscles is highly induced by fatty acids (63, 64, 65). In both tissues, ANGPTL4 functions as a local inhibitor of LPL and is part of a fatty acid- and PPARδ-activated feedback mechanism that controls the uptake of fatty acids to avoid lipid overload (63, 66, 67). ANGPTL4 does not seem to influence the protein abundance of LPL in the heart (45).

In the human skeletal muscles, ANGPTL4 likely plays a role in plasma lipid partitioning between active and nonactive muscles (66). During exercise, ANGPTL4 is induced via elevated nonesterified fatty acids and PPARδ in the nonactive muscles, reducing the local uptake of plasma TG-derived fatty acids. In the working muscles, this mechanism is countered by 5' adenosine monophosphate-activated protein kinase-mediated suppression of ANGPTL4 expression to promote the use of plasma TG as a fuel for active muscles (66). Consistent with the role of muscle ANGPTL4 in local regulation of LPL, the deficiency of ANGPTL4 increases LPL activity in skeletal muscles of mice (68).

### Role of ANGPTL4 in macrophages

Cultured macrophages produce and constitutively secrete LPL and ANGPTL4 (69, 70). The expression of ANGPTL4 in macrophages is induced by various types of lipids, including fatty acids, acetylated LDL (acLDL), oxidized LDL (oxLDL), and natural and synthetic TG emulsions (43, 69, 71, 72). The addition of recombinant ANGPTL4 as well as the overexpression of ANGPTL4 decrease LPL activity and impair the uptake of oxLDL/acLDL and TG emulsions in macrophages, thereby mitigating foam cell formation and lipid-induced cell stress (69, 72, 73). By contrast, ANGPTL4 deficiency enhances the uptake of oxLDL/acLDL and TG emulsions in macrophages and increases lipid-induced cell stress and apoptosis (43, 71). In agreement with these data, inactivation of ANGPTL4 in mice fed a Western-type diet leads to foam cell formation in the mesenteric lymph nodes and promotes atherosclerosis (40, 71, 72, 74). Taken together, by repressing LPL activity, ANGPTL4 functions as an important regulator of lipid uptake in macrophages. Unlike in adipocytes, ANGPTL4 does not regulate LPL mass in macrophages (43).

## Physiological role of circulating ANGPTL4

The presence of ANGPTL4 in the human blood plasma has been studied using ELISA and Western blot. Two different ELISAs exist, one of which detects full-length ANGPTL4 and the C-terminal ANGPTL4 fragment (64, 75), whereas the other ELISA detects full-length and N-terminal ANGPTL4 (16). Western blots performed on human plasma have specifically measured the N-terminal ANGPTL4 fragment (58). Integrating and extrapolating the results of various studies that have measured ANGPTL4 in human plasma points to the following scenario (16, 30, 58, 64, 75).

Most of the ANGPTL4 in human plasma represents the C-terminal ANGPTL4 fragment. This fragment does not inhibit LPL, in contrast to full-length or N-terminal LPL. The total concentration of N-terminal ANGPTL4 in plasma is higher than the total concentration of full-length ANGPTL4 (58). There is very little full-length and N-terminal ANGPTL4 in human plasma in free form. Most of the circulating full-length and N-terminal ANGPTL4 is complexed with ANGPTL8 (16). The origin of circulating full-length and N-terminal ANGPTL4 is not fully understood, but it is likely that adipose tissue and liver are the primary sources, respectively (58). Because binding of ANGPTL8 strongly disables the ability of ANGPTL4 to inhibit LPL, and because C-terminal ANGPTL4 is unable to inhibit LPL, this scenario suggests that the circulating forms of ANGPTL4 may not be involved in LPL regulation. A counterargument that could be raised is that intravenous injection of recombinant full-length ANGPTL4 raises plasma TG levels (11). However, it is unclear whether this result reflects the physiological role of ANGPTL4.

With respect to regulation, plasma levels of C-terminal ANGPTL4 increase whenever the levels of nonesterified fatty acids increase (65), including during fasting, hypocaloric diets, and exercise (30, 66, 75). In contrast, the plasma levels of C-terminal ANGPTL4 are decreased by insulin infusion and are also lower in E40K carriers (76). Plasma levels of N-terminal ANGPTL4 are also increased during fasting (30). Interestingly, the plasma levels of C- and N-terminal ANGPTL4 are increased by fenofibrate, which specifically targets the hepatic production of ANGPTL4 (58, 77). Furthermore, fenofibrate raises plasma levels of human C-terminal ANGPTL4 in hepatocyte-humanized mice (78). These data suggest that a major portion of circulating ANGPTL4 is liver-derived.

## Biochemical mechanism of LPL inhibition by ANGPTL4

Inhibition of LPL by ANGPTL4 was initially attributed to the dissociation of catalytically active LPL dimers into inactive LPL monomers (79). However, whether LPL is only active as a dimer has been called into question (80). More recent evidence indicates that ANGPTL4 binds to regions proximal to LPL’s catalytic pocket (81), and that ANGPTL4 promotes the irreversible unfolding of this hydrolase domain (82, 83, 84). Such a catalytic action of ANGPTL4 reportedly does not require the conversion of LPL homodimers into monomers (83) but renders LPL more prone to cleavage by PCSK3 (47). The binding and inhibition of LPL by ANGPTL4 are mediated by a stretch of amino acids located in the N-terminal region of ANGPTL4 that contains the polar residues His46, Gln50, and Gln53 (85, 86).

As an alternative to promoting irreversible LPL unfolding, it has been suggested that ANGPTL4 functions as a reversible, noncompetitive inhibitor of LPL (87). By binding close to the catalytic site of LPL, ANGPTL4 is supposedly able to prevent substrate catalysis at the active site (81). How such a mechanism could trigger the cleavage and degradation of LPL in adipocytes is unclear.

In addition to promoting the intracellular cleavage and degradation of LPL, ANGPTL4 is also able to inhibit extracellular, endothelial-bound LPL. However, the potency of ANGPTL4 to inhibit and unfold LPL is lower when LPL is bound to GPIHBP1 due to the LPL-stabilizing action of GPIHBP1 (82, 88). Stabilization of LPL is mediated by the N-terminal domain of GPIHBP1, an intrinsically disordered region rich in acidic residues.

## Impact of ANGPTL4 on plasma cholesterol

**Fig. 2:**
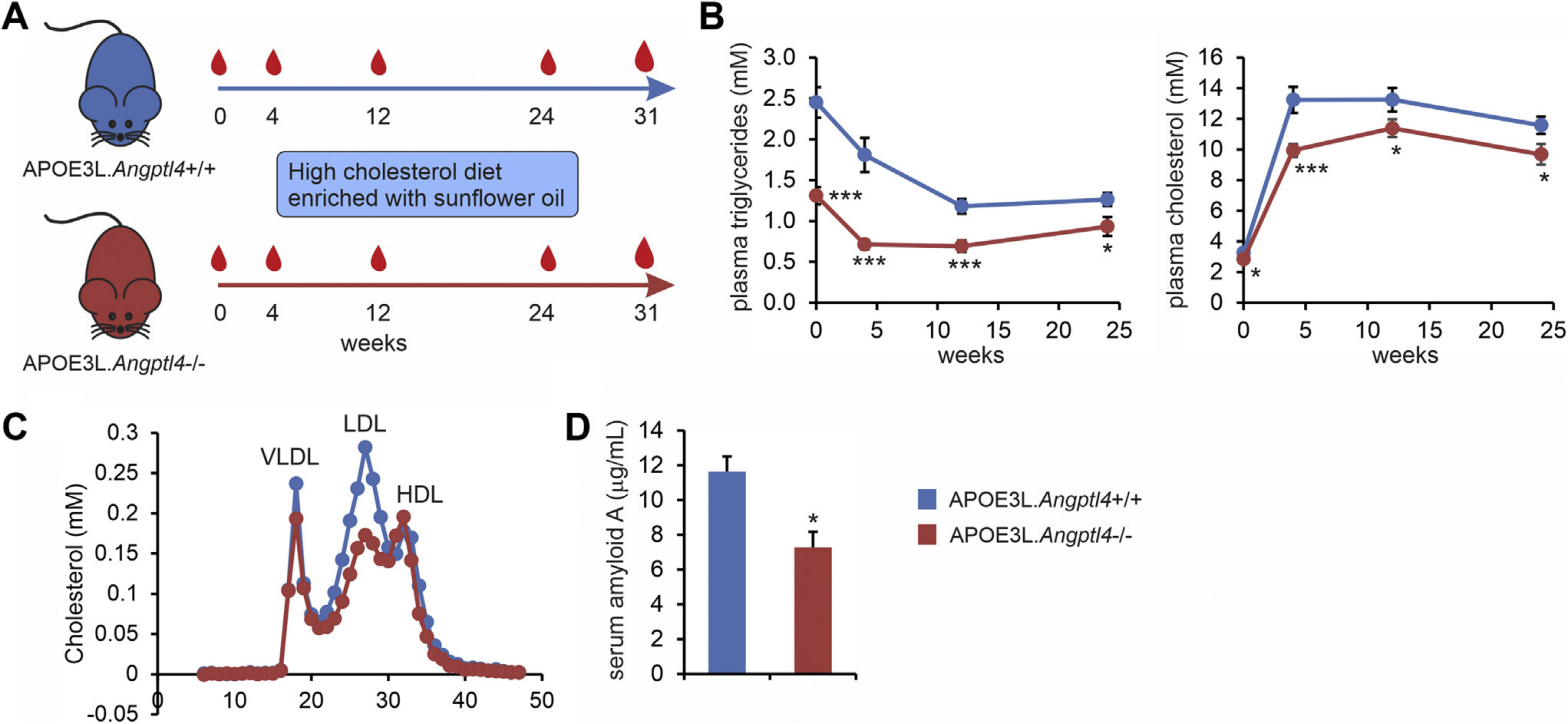
Lower plasma LDL-C in hypercholesterolemic ANGPTL4-deficient mice. A: Schematic description of the study. At 10–15 weeks of age, 18 female ANGPTL4-deficient mice on an APOE3Leiden background and 18 female control APOE3Leiden mice were fed a Western-type diet rich in cholesterol (0.4%) and sunflower oil (33 energy%) for 31 weeks. B: Plasma triglycerides and plasma cholesterol in tail bleeds. C: FPLC-based lipoprotein profiling on pooled plasma collected at the final bleed. D: Serum-amyloid A levels in plasma collected at the final bleed. The error bars are SEM. N = 15–18 per group. Asterisk indicates significantly different from control mice according to Student’s *t* test (∗*P* < 0.05, ∗∗∗*P* < 0.001). ANGPTL, angiopoietin-like protein.

Evidence abounds indicating that ANGPTL4 impacts plasma TG levels in mice and humans. By contrast, the evidence linking ANGPTL4 to the regulation of plasma LDL-C is much more limited. The whole-body ANGPTL4 deficiency in LDLR-deficient mice fed a Western-type diet was associated with a marked decrease in plasma cholesterol (71). However, these results are likely confounded by the lymphadenopathy, ascites, and peritonitis in the ANGPTL4-deficient mice (71). In studies in mice that were approved by the Institutional Animal Use and Care Committee of Wageningen University (2011063.c), we found that the whole-body ANGPTL4 deficiency in hypercholesterolemic APOE3Leiden mice fed a high cholesterol-high unsaturated fat diet was associated with a significant decrease in plasma TG and total cholesterol (Fig. 2A, B), the latter of which was mainly explained by a marked reduction in LDL-C levels (Fig. 2C). The ANGPTL4-deficient APOE3Leiden mice did not show any lymphadenopathy, ascites, and peritonitis, and exhibited decreased rather than increased serum amyloid A levels (Fig. 2D), suggesting that the lower cholesterol levels are not secondary to a complex pathological phenotype. Recently, it was shown in PCSK9-induced hypercholesterolemic mice that liver-specific deficiency of ANGPTL4 is also associated with a significant decrease in plasma total cholesterol levels, which was accounted for by reductions in plasma VLDL-C and IDL/LDL-C (57). Overall, these data suggest that in hypercholesterolemic mice, the liver or whole-body ANGPTL4 deficiency leads to a decrease in plasma LDL-C. Presently, the mechanism underlying the effect of ANGPTL4 on LDL-C levels remains unclear.

Although studies in mice thus suggest a role of ANGPTL4 in regulating plasma LDL-C levels, human genetic studies do not support this notion. Specifically, LOF mutations in ANGPTL4 are associated with lower plasma TG and higher plasma HDL-C levels but, unlike LOF mutations in ANGPTL3 levels (89, 90), are not associated with a significant change in plasma LDL-C levels (20, 21, 22). How it is possible that the studies in ANGPTL4-deficient mice and the human genetic studies lead to different conclusions is unclear.

As alluded to above, plasma HDL-C levels are elevated in the carriers of LOF mutations in ANGPTL4. The higher HDL-C may be secondary to enhanced VLDL lipolysis, thereby reducing CETP-mediated cholesterol transfer from HDL to VLDL. Interestingly, it was recently suggested that ANGPTL4 can inhibit endothelial lipase in vitro (91), which is expected to translate into lower rather than higher plasma HDL-C levels in ANGPTL4 LOF carriers. Studies in mice have not provided uniform results on the impact of ANGPTL4 on HDL-C. Specifically, the whole-body ANGPTL4 deficiency was associated with higher HDL-C levels (42), adipocyte-specific ANGPTL4 deficiency was not associated with any change in HDL-C (92), and hepatocyte-specific ANGPTL4 deficiency was associated with slightly lower HDL-C levels (57). Plasma HDL-C levels were minimally changed in ANGPTL4-deficient APOE3Leiden mice (Fig. 2C). Overall, whether ANGPTL4 directly regulates plasma HDL-C levels, possibly by inhibiting endothelial lipase in vivo, requires further investigation.

## Future perspectives and conclusion

In the last few years, major progress has been made in elucidating the (tissue-specific) role of ANGPTL4 in governing plasma lipids. Currently, the important outstanding questions on ANGPTL4 are as follows:

To what extent is the effect of liver-specific inactivation of ANGPTL4 on plasma TG dependent on fasting? Does liver-specific inactivation of ANGPTL4 on top of ANGPTL3 inactivation provides an additional therapeutic benefit by further lowering of plasma lipids? Does liver-specific inactivation of ANGPTL4 impact plasma lipids in humans? Does liver-derived ANGPTL4 form a complex with ANGPTL8? What is the exact mechanism by which ANGPTL8 interferes with LPL inhibition by ANGPTL4 in adipose tissue? How does ANGPTL4 raise plasma LDL-C levels in hypercholesterolemic mice, and are these data of any relevance to humans? Does ANGPTL4 produced by tissues other than liver and adipose tissue influence plasma TG levels? Future studies should be directed toward answering these questions.

In conclusion, although for many years ANGPTL4 was dismissed as a pharmacological target, the recent mouse studies indicate that liver-specific inactivation of ANGPTL4 may be a viable strategy for lowering plasma TG and possibly LDL-C. Clinical trials supporting the lipid-lowering effects of ANGPTL4 inactivation are eagerly awaited. In the future, anti-ANGPTL4 therapies, either as monotherapy or coupled to anti-ANGPTL3 therapies, may become a treatment option for reducing plasma lipids in selected patient groups.

## Conflict of interest

The author declares that he has no conflicts of interest with the contents of this article.
